# Unplanned readmission or death after discharge for Aboriginal and non-Aboriginal people with chronic disease in NSW Australia: a retrospective cohort study

**DOI:** 10.1186/s12913-018-3723-4

**Published:** 2018-11-26

**Authors:** Amanda Jayakody, Christopher Oldmeadow, Mariko Carey, Jamie Bryant, Tiffany Evans, Stephen Ella, John Attia, Rob Sanson-Fisher

**Affiliations:** 1 Health Behaviour Research Collaborative, School of Medicine and Public Health, Faculty of Health and Medicine, University of Newcastle, Callaghan, NSW 2308 Australia; 2 Priority Research Centre for Health Behaviour, University of Newcastle, Callaghan, NSW Australia; 3 Hunter Medical Research Institute, New Lambton Heights, NSW Australia; 4 CREDITSS—Clinical Research Design, Information Technology and Statistical Support Unit, Hunter Medical Research Institute, HMRI Building, New Lambton Heights, NSW Australia; 5 School of Medicine and Public Health, University of Newcastle, Callaghan, NSW 2308 Australia; 6 Nunyara Aboriginal Health Unit, Central Coast Local Health District, Ward Street, Gosford, NSW Australia

**Keywords:** Aboriginal health, Unplanned readmission, Health services research, Data linkage, Chronic disease

## Abstract

**Background:**

Admitted patients with chronic disease are at high risk of an unplanned hospital readmission, however, little research has examined unplanned readmission among Aboriginal people in Australia. This study aimed to examine whether rates of unplanned 28 day hospital readmission, or death, significantly differ between Aboriginal and non-Aboriginal patients in New South Wales, Australia, over a nine-year period.

**Methods:**

A retrospective cohort analysis of a sample of de-identified linked hospital administrative data was conducted. Eligible patients were: 1) aged ≥18 years old, 2) admitted to an acute facility in a NSW public hospital between 30th June 2005 and 1st July 2014, and 3) admitted with either cardiovascular disease, chronic respiratory disease, diabetes or renal disease. The primary composite outcome was unplanned readmission or death within 28 days of discharge. Generalized linear models and a test for trend were used to assess rates of unplanned readmission or death over time in Aboriginal and non-Aboriginal patients with chronic disease, accounting for sociodemographic variables.

**Results:**

The final study cohort included 122,145 separations corresponding to 48,252 patients (Aboriginal = 57.2%, *n* = 27,601; non-Aboriginal = 42.8%, *n* = 20,651). 13.9% (*n* = 16,999) of all separations experienced an unplanned readmission or death within 28 days of discharge. Death within 28 days of discharge alone accounted for only a small number of separations (1.4%; *n* = 1767). Over the nine-year period, Aboriginal separations had a significantly higher relative risk of an unplanned readmission or death (Relative risk = 1.34 (1.29, 1.40); *p*-value < 0.0001) compared with non-Aboriginal separations once adjusted for sociodemographic, disease variables and restricted to < 75 years of age. A test for trend, including an interaction between year and Aboriginal status, showed there was no statistically significant change in proportions over the nine-year period for Aboriginal and non-Aboriginal separations (*p*-value for trend = 0.176).

**Conclusion:**

Aboriginal people with chronic disease had a significantly higher risk of unplanned readmission or death 28 days post discharge from hospital compared with non-Aboriginal people, and there has been no significant change over the nine year period. It is critical that effective interventions to reduce unplanned readmissions for Aboriginal people are identified.

**Electronic supplementary material:**

The online version of this article (10.1186/s12913-018-3723-4) contains supplementary material, which is available to authorized users.

## Background

On average Aboriginal and Torres Strait Islander people (Aboriginal people hereafter)^1^ experience, on average, a 10 year gap in life expectancy compared with non-Aboriginal Australians. Two thirds of this gap is accounted for by chronic disease [1]. Chronic diseases in Aboriginal people are both more prevalent and occur at a much younger age[1, 2]. Aboriginal people have higher self-reported rates of cardiovascular disease, respiratory disease, diabetes and renal disease than non-Aboriginal people [1, 2].

Although most chronic diseases should ideally be managed in the community health setting, admissions to hospital related to chronic disease are common and represent the largest proportion of potentially avoidable hospitalisations [3]. Factors such as poor discharge planning, poor community follow up from health care services, and a lack of support for the patient and carer in chronic disease self-management skills mean that many hospital admissions for chronic disease are followed by an unplanned hospital readmission [4–8]. Unplanned readmissions are defined as admissions to hospital which were not planned and which usually occur within one month of discharge from an initial (i.e. index) admission [9, 10]. Unplanned readmissions are a financial burden to the health system, and cause an emotional and time burden on patients and their families [11, 12]. Admitted patients with chronic disease are known to be at high risk of an unplanned hospital readmission, with readmission highest amongst patients with cardiovascular disease, respiratory disease and diabetes [11, 13, 14]. In the Australian state of New South Wales (NSW) 13% of patients with COPD and 9% of patients with CHF were readmitted within 28 days [3]. In Australia, unplanned readmissions are considered an indicator relating to “high quality and affordable hospital and hospital related care” in the Australian National Healthcare Agreement, and unplanned readmissions are included in the NSW service performance indicators to provide a mechanism for monitoring and managing the performance of hospitals [9, 15].

However there is limited knowledge of the rate of unplanned readmission for Aboriginal people with chronic disease. In a NSW Chief Health Officer’s report on the health of Aboriginal people of NSW, the all-cause (all medical and surgical) unplanned readmission rate within 28 days for Aboriginal people was 8.1% (compared with 6.3% for non-Aboriginal people) [9]. The all-cause readmission rate has remained consistently higher for Aboriginal people [9]. However little is known regarding the patterns over time for unplanned readmissions amongst Aboriginal people with chronic disease. An analysis of readmission rates within one regional western NSW hospital found the proportion of Aboriginal patients readmitted to hospital had increased from 11.7% in 1996 to 18.3% in 2005, however there was no significant trend over time [16]. This analysis did not look specifically at trends in chronic diseases for Aboriginal patients, and the data may not be representative of NSW Aboriginal people as a whole.

Given the high burden of chronic disease and high rates of unplanned readmission rates among Aboriginal people, there is a need for more specific analysis of unplanned readmissions related to chronic disease in order to identify potential differences and patterns amongst Aboriginal and non-Aboriginal people over time. The purpose of our study was to examine amongst Aboriginal and non-Aboriginal people with chronic disease in NSW from 2005/6 to 2014/15: 1) whether the proportion of separations with an unplanned 28 day readmission or death significantly differ between Aboriginal and non-Aboriginal patients; 2) the extent to which sociodemographic, disease and separation factors are associated with any differences; and 3) how the proportion of separations with an unplanned 28 day hospital readmission or death changed over the nine-year period.

## Methods

### Ethics approval

The study was approved by the NSW Population & Health Services Research Ethics Committee (HREC/15/CIPHS/18) and the NSW Aboriginal Health and Medical Research Council Ethics Committee (1090/15).

### Study design and data sources

This study was a retrospective cohort analysis of de-identified linked hospital administrative data. The linked data were derived from three datasets:

1. NSW Admitted Patient Data Collection (APDC): the APDC contains records of all admitted patient services provided by NSW public hospitals, private hospitals/centres and psychiatric hospitals.
2. NSW Emergency Department Data Collection (EDDC): the EDDC contains records for patient presentations to emergency departments in NSW public hospitals.
3. NSW Registrar of Births, Deaths and Marriages (RBDM): the RBDM contains mortality information for the NSW population.

### Study sample

#### Eligibility criteria

**Fig. 1:**
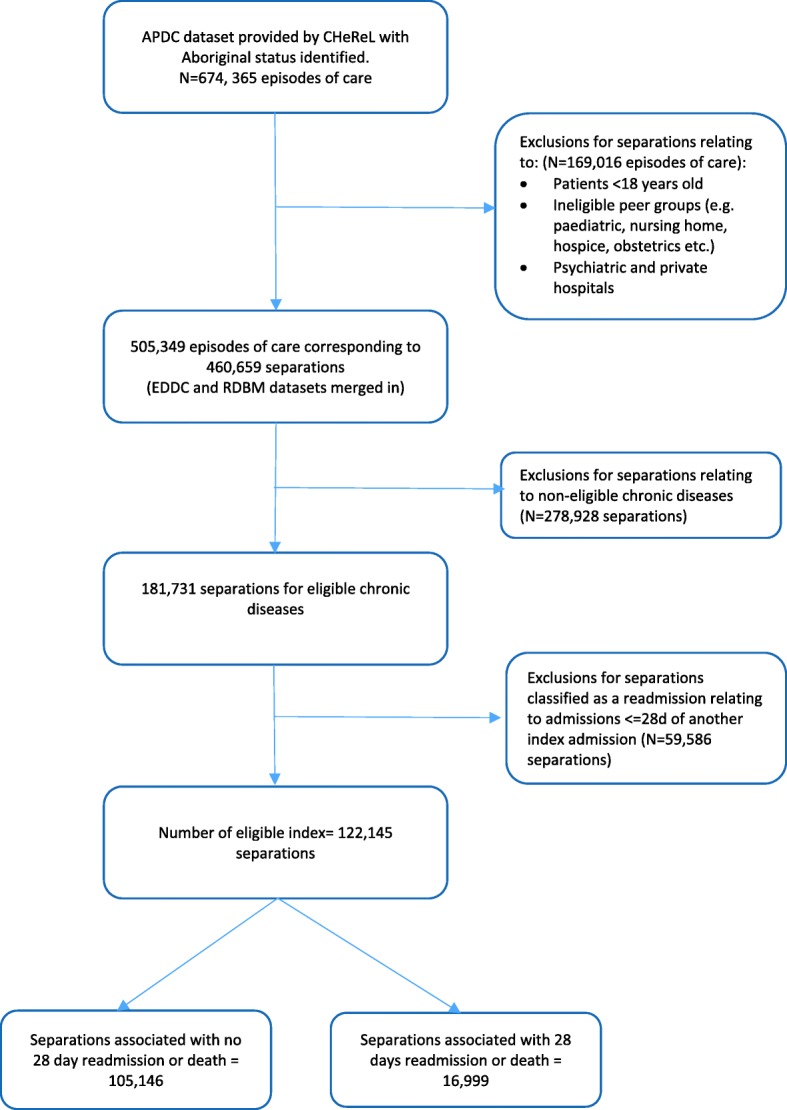
Flow diagram of dataset generation

The study sample included patients who were: 1) aged 18 years and older at the time of admission; 2) admitted to an acute facility in a NSW public hospital between 30th June 2005 and 1st July 2014; 3) discharged from hospital to the community; and 4) had one or more of the following ICD-10 defined chronic diseases as a principle or additional diagnosis: cardiovascular disease, diabetes, respiratory disease and renal diseases (See Additional file 1 for a list of ICD-10 codes). Figure 1 outlines how the dataset was generated.

### Sampling

All patients meeting the eligibility criteria who had at least one APDC separation in the period of interest, and where status was recorded as Aboriginal and/or Torres Strait Islander person on any APDC record were selected. In order to obtain a comparison sample of non-Aboriginal persons, a sampling frame was then generated consisting of a list of patients meeting the eligibility criteria recorded in the APDC, excluding the list of Aboriginal persons obtained above. A random sample of person identification numbers (of the same number as the Aboriginal cases) was selected from the sampling frame, forming the non-Aboriginal patient sample. These patients had no APDC records with Aboriginality coded as ‘yes’. EDDC and RBDM death records which linked to the patients were extracted and included in the final sample.

### Data linkage

The data sources were linked by the Centre for Health Record Linkage using probabilistic record linkage methods [17]. All data were provided in a de-identified format. The data were supplied as episodes of care. Each episode of care ends with a statistical discharge; each statistical discharge occurs due to discharge, death, transfer, or change of care type.

### Data cleaning

Duplicate records were excluded. Separations were defined by combining nested, overlapping and contiguous episodes of care creating periods for which patients were hospitalised. Therefore separations are defined as the total hospital stay (from admission to discharge from hospital). For our analyses we retained the diagnosis codes and admission data from the first episode of each separation, but our discharge date, from which 28 day readmission or death is considered, was the latest discharge date for the period of hospitalisation. The unit of analysis was separations.

### Variables

*Primary outcome:* The primary composite outcome was all-cause unplanned hospital readmission or death within 28 days of separation from any acute facility in a NSW public hospital. An unplanned readmission is defined as occurring within 28 days of discharge from an initial (i.e. index) admission. ‘Unplanned’ refers to separations coded as an ‘emergency status recode’ in the APDC. Readmissions due to mental health, cancer, hospital in home care, chemotherapy or dialysis were excluded. Separations were excluded if death occurred during admission or if the patient was discharged to palliative care. Sensitivity analyses were conducted to examine the effect of deaths or discharge to palliative care during admission which is described in the statistical analysis section. Each subsequent separation that fell outside of the 28 day timeframe was counted as a new index separation (see Fig. 1). All-cause readmission or death was calculated as follows: Numerator: total number of 28 day unplanned readmission or death for any cause associated with an eligible index admission. Denominator: number of admissions with an included chronic disease (principal or additional diagnosis) and an index admission.

#### Explanatory variables

The following explanatory variables correspond to those recorded at the beginning of each separation.

1. Sociodemographic variables: Patient’s gender, age, Aboriginal status and marital status. The Accessibility/Remoteness Index of Australia (ARIA) and the Index of Relative Socio-economic Disadvantage (IRSD) quintile were also included. ARIA is the standard Australian Bureau of Statistics (ABS) endorsed measure of remoteness and is derived from measures of road distances between populated localities and service centres [18]. The IRSD is a general socio-economic index that summarises a range of information about the economic and social conditions of people and households within a geographic area [19].
2. Disease-related variables: the Charlson Co-morbidity Index (CCI) was included [20]. The CCI is an index of the risk of mortality from comorbidity during the next 12 months and calculates a score from secondary diagnoses of admissions weighted for type of condition. The CCI scores were dichotomised into three groups of 0, 1 and 2 or more. Zero indicating the lowest score and 2 or more indicating the highest scores.
3. Separation variables: The following variables were included for each hospital separation: financial year of separation and length of stay (days).

#### Statistical analysis

Chi-square and t-tests were used to examine crude associations between Aboriginal status and sociodemographic, disease and separation factors. A log-binomial generalised linear model (GLM) was used to determine the association between Aboriginal status and unplanned readmission or death over the nine year period, and then restricted to patients aged ≤75 years due to the differential age structures between Aboriginal and non-Aboriginal patients. Exponentiated parameter estimates from this model (interpreted as relative risks) are presented together with 95% confidence intervals and *p*-values. A propensity score analysis was conducted to account for potential selection bias due to differences in the probability of dying during admission to hospital between Aboriginal and non-Aboriginal people [21]. The propensity score was estimated using a logistic regression model (death in hospital or discharged to palliative care as the outcome, sociodemographic, disease and separation factors as predictors), and stabilised propensity scores were used as weights (inverse probability of “treatments”) in the GLM. Unplanned readmission or death within 28 days trends over the study period were assessed by including a term for financial year (as a continuous variable) in the GLM, as well as an interaction term between Aboriginal status and year, which assessed differences in the trends by Aboriginal status. The model was adjusted for sociodemographic and disease variables. A sensitivity analysis was conducted to examine any potential differences in results obtained using an all-cause compared to a chronic disease specific readmission rate. All analyses used Stata V.11.2 [22].

## Results

In the linked dataset there were 674, 365 hospital episodes of care for Aboriginal and non-Aboriginal patients. After separations not meeting inclusion criteria were excluded, the final study cohort included 122,145 separations (Aboriginal = 77,427; Non-Aboriginal = 44,718), corresponding to 48,252 patients (Aboriginal = 57.2%, *n* = 27,601; non-Aboriginal = 42.8%, *n* = 20,651). Table 1 describes the characteristics of separations by Aboriginal status. Aboriginal separations were significantly younger and had a higher proportion of female separations, compared with non-Aboriginal separations. A smaller proportion of Aboriginal separations corresponded to individuals who were married or in a de facto relationship compared with non-Aboriginal separations. Compared to non-Aboriginal separations, there were a higher proportion of separations associated with diabetes and chronic respiratory disease among Aboriginal separations. Cardiovascular disease was significantly higher amongst non-Aboriginal separations and is evidenced in the higher Charlson comorbidity index which gives greater weight to cardiovascular disease. A higher proportion of Aboriginal separations correspond to individuals residing in the most disadvantaged geographic and remote/very remote areas of NSW. Aboriginal separations had a lower average length of stay compared with non-Aboriginal separations.

**Table 1:** Characteristics of separations by Aboriginal status (*n* = 122,145)

		Aboriginal (*n* = 77,427) *n* (%)	Non-Aboriginal (*n* = 44,718) *n* (%)	*p*-value
Sex	% Female	42,982 (55.5)	22,422 (50.1)	> 0.001
Age	Mean (SD)	53.5 (16.5)	66.7 (17.9)	> 0.001
Marital status	Married/de facto	30,992 (40.1)	23,815 (53.3)	> 0.001
	Single	25,178 (32.5)	5673 (12.7)	
	Widowed	9385 (12.1)	9884 (22.1)	
	Divorced/separated	10,434 (13.5)	4720 (10.6)	
	Not known	1372 (1.8)	592 (1.3)	
Chronic diseases present at admission	Diabetes	32,865 (39.5)	11,853 (26.5)	> 0.001
	Chronic respiratory disease	15,403 (19.9)	6135 (13.7)	< 0.0001
	Cardiovascular disease	41,977 (54.2)	29,231 (65.4)	< 0.0001
	Renal disease	20,133 (26.0)	12,638 (28.3)	< 0.0001
Charlson Co-morbidity Index score	0	43,888 (56.7)	25,454 (56.9)	< 0.0001
	1	18,835 (24.3)	9979 (22.3)	
	2+	14,704 (19.0)	9285 (20.8)	
IRSD	1st quintile - most disadvantaged	19,505 (25.2)	5823 (13.0)	< 0.0001
	2nd quintile	22,584 (29.2)	10,529 (23.6)	
	3rd quintile	16,701 (21.6)	8788 (19.7)	
	4th quintile	14,286 (18.5)	9985 (22.3)	
	5th quintile - least disadvantaged	4351 (5.6)	9593 (21.4)	
ARIA	Highly Accessible (major cities)	29,855 (38.6)	31,521 (70.5)	< 0.0001
	Accessible (inner regional)	29,132 (37.6)	10,348 (23.1)	
	Moderately Accessible (outer regional)	13,692 (17.7)	2560 (5.7)	
	Remote / Very Remote	4748 (6.1)	289 (0.7)	
Year of separation	2005–06	7547 (10.3)	4680 (11.2)	< 0.0001
	2006–07	7840 (10.7)	4719 (11.3)	
	2007–08	7980 (10.9)	4693 (11.2)	
	2008–09	6905 (9.5)	4143 (9.9)	
	2009–10	7041 (9.6)	4169 (10.0)	
	2010–11	7010 (9.6)	4061 (9.7)	
	2011–12	7807 (10.7)	4343 (10.4)	
	2012–13	10,243 (14.0)	5391 (12.9)	
	2013–14	10,643 (14.6)	5644 (13.5)	
Length of stay (days)	Mean (SD)	5.6 (14.2)	6.9 (17.2)	< 0.0001

13.9% (*n* = 16,999) of all separations experienced an unplanned readmission or death within 28 days of discharge. Death within 28 days of discharge accounted for only a small number of separations overall (1.4%; *n* = 1767). An unadjusted regression, demonstrated that Aboriginal separations had a significantly higher risk of an unplanned readmission or death within 28 days of discharge compared with non-Aboriginal separations (Table 2; Relative risk (RR) = 1.16; 95% confidence intervals (CI):1.13, 1.19; *p*-value: < 0.0001). To account for the younger age distribution in Aboriginal people compared with non-Aboriginal people, the model was restricted to people aged < 75 years old. This resulted in the relative risk increasing to 1.36 (95% CI:1.30, 1.41; *p*-value: < 0.0001). A sensitivity analysis, was conducted to examine any potential differences between using an all-cause compared to a chronic disease specific readmission rate, and results were broadly similar.

**Table 2:** Differences in separations that resulted in an unplanned readmission or death by Aboriginal status, for the period 2005/6–2013/14

	Unplanned readmission or death (n = 122,145)		Unadjusted relative risk (RR), (95% CI; *P*-value)	RR restricted to patients aged < 75 yrs., (95% CI; *P*-value)
	Yes	No		
Aboriginal *n* (%)	11,349 (14.7)	66,078 (85.3)	1.16 (1.13, 1.19; < 0.0001)	1.36 (1.30, 1.41; < 0.0001)
Non-Aboriginal *n* (%)	5650 (12.6)	39,068 (87.4)	ref	ref

### Propensity score weighted analyses

Aboriginal people were significantly less likely to die during admission or be discharged to palliative care compared with non-Aboriginal people (Odds ratio = 0.73; 95% CI: 0.68, 0.79; < 0.001; AUC = 0.7714; pseudo R2 = 0.1096). Sample weights were created using a stabilised propensity score to account for the potential selection bias due to the difference in probability of dying during admission. The propensity score sample weight was included in the following regression analyses whilst separations which ended in death during admission or the patient being discharged to palliative care were excluded from the analysis (2.84%, *n* = 3570).

### Adjusted regression analyses

Table 3 shows the unadjusted and adjusted regression models examining the effect of sociodemographic and disease variables on the association of Aboriginal status and unplanned readmission or death. Aboriginal separations continued to have a significantly higher risk of an unplanned readmission or death compared with non-Aboriginal separations once adjusted for sociodemographic (including the age restriction) and disease variables, including the propensity score sampling weight (RR = 1.34; CI:1.29, 1.40; *p* < 0.0001). Length of stay was not included in the final model because of the direction of its relationship with Aboriginal status and readmission. Although it was associated with both Aboriginal status and readmission, a sensitivity analysis including length of stay in the adjusted model showed that the overall results were broadly similar. Apart from financial year, all sociodemographic and disease variables remained significantly associated with readmission after controlling for all variables in the table.

**Table 3:** Unadjusted and adjusted GLM regression models of unplanned readmission or death by Aboriginal status for the study period 2005/6 to 2013/14

	Unplanned readmission or death Relative risks (RR) (95% CI)					
	Unadjusted RR with propensity score (PS) weight	Unadjusted RR without PS weight	Adjusted^a^ RR with PS weight	Adjusted^a^ RR without PS weight	Adjusted^a^ RR with PS weight and restricted to < 75 years	*P*-value
Aboriginal status						
Non-Aboriginal	ref	ref	ref	Ref	red	
Aboriginal	1.15 (1.12, 1.19)	1.16 (1.13, 1.20)	1.29 (1.24, 1.33)	1.29 (1.24, 1.33)	1.34 (1.29, 1.40)	< 0.0001
Year	1.00 (0.99, 1.01)	1.00 (0.99, 1.01)	1.00 (0.99, 1.01)	1.00 (0.99, 1.01)	1.00 (0.99, 1.01)	0.529
Gender	–	–	–			
Male	–	–	ref	ref	ref	
Female	–	–	0.89 (0.86, 0.92)	0.89 (0.86, 0.91)	0.87 (0.84, 0.90)	< 0.0001
Age	–	–	1.01 (1.01, 1.01)	1.01 (1.01, 1.01)	1.01 (1.00, 1.01)	< 0.0001
Marital status	–	–				
Married	–	–	ref	ref	ref	
Single	–	–	1.19 (1.15, 1.24)	1.20 (1.15, 1.24)	1.20 (1.15, 1.25)	< 0.0001
Widowed	–	–	1.18 (1.13, 1.23)	1.18 (1.13, 1.23)	1.21 (1.13, 1.28)	< 0.0001
Divorced/separated	–	–	1.15 (1.10, 1.20)	1.15 (1.10, 1.20)	1.18 (1.12, 1.24)	< 0.0001
Not known	–	–	0.92 (0.81, 1.05)	0.93 (0.82, 1.06)	0.97 (0.84, 1.12)	0.685
IRSD						
1st quintile - most disadvantaged	–	–	ref	ref	ref	
2nd quintile	–	–	0.94 (0.90, 0.98)	0.94 (0.90, 0.98)	0.94 (0.90, 0.99)	< 0.05
3rd quintile	–	–	0.94 (0.90, 0.99)	0.94 (0.90, 0.99)	0.93 (0.88, 0.98)	< 0.05
4th quintile	–	–	0.93 (0.88, 0.98)	0.93 (0.88, 0.98)	0.94 (0.88, 0.99)	< 0.05
5th quintile – least disadvantaged	–	–	0.88 (0.83, 0.94)	0.88 (0.83, 0.94)	0.80 (0.74, 0.87)	< 0.0001
ARIA						
Highly Accessible	–	–	ref	ref	ref	
Accessible	–	–	0.93 (0.89, 0.96)	0.93 (0.89, 0.96)	0.93 (0.90, 0.97)	> 0.01
Moderately Accessible	–	–	0.87 (0.84, 0.93)	0.87 (0.84, 0.93)	0.87 (0.82, 0.92)	< 0.0001
Remote/Very Remote	–	–	0.67 (0.61, 0.73)	0.67 (0.61, 0.73)	0.65 (0.59, 0.72)	< 0.0001
Charlson Index score						
0	–	–	ref	ref	ref	
1	–	–	1.43 (1.38, 1.48)	1.43 (1.38, 1.48)	1.45 (1.39, 1.51)	< 0.0001
2+	–	–	1.63 (1.57, 1.69)	1.63 (1.57, 1.69)	1.69 (1.62, 1.77)	< 0.0001

^a^RRs are adjusted for all variables given in the table

**Fig. 2:**
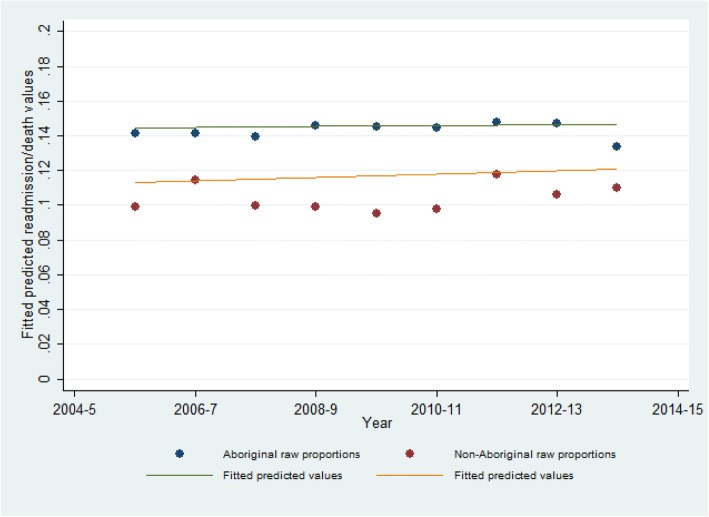
Trend analysis for unplanned readmission or death calculated for each year of the study period (2005–6 to 2013–14) by Aboriginal status

Figure 2 displays the raw proportions and predicted probabilities (obtained from the final GLM model shown in Table 4) of unplanned readmission or death by Aboriginal status. There was no statistically significant change in the proportion of separations that resulted in an unplanned readmission or death over the nine-year period for Aboriginal and non-Aboriginal separations (*p*-value for trend = 0.176). The apparent gap between the fitted values and raw proportions are due to the fact that the fitted values are adjusted for sociodemographic variables.

**Table 4:** Testing for a trend over time in unplanned readmission or death: Unadjusted and adjusted GLM regression models of unplanned readmission or death by Aboriginal status including an interaction term for year and Aboriginal status (2005/6 to 2013/14)

	Unplanned readmission or death with interaction term Relative risks (RR) (95% CI)					
	Unadjusted RR with propensity score (PS) weight	Unadjusted RR without PS weight	Adjusted^a^ RR with PS weight	Adjusted^a^ RR without PS weight	Adjusted^a^ RR with PS weight and restricted to < 75 years	*P*-value
Aboriginal status						
Non-Aboriginal	ref	ref	ref	Ref	red	
Aboriginal	1.15 (1.11, 1.19)	1.16 (1.12, 1.20)	1.28 (1.23, 1.32)	1.28 (1.23, 1.33)	1.33 (1.27, 1.39)	< 0.0001
Year	1.00 (0.99, 1.01)	1.00 (0.99, 1.01)	1.00 (0.99, 1.01)	0.99 (0.99, 1.01)	1.01 (0.99, 1.02)	0.140
Interaction term						
Year and Aboriginal status	1.00 (0.99, 1.02)	1.01 (0.99, 1.02)	1.01 (0.99, 1.02)	1.01 (1.00, 1.02)	0.99 (0.97, 1.00)	0.176
Gender	–	–	–			
Male	–	–	ref	ref	ref	
Female	–	–	0.89 (0.86, 0.92)	0.89 (0.86, 0.92)	0.88 (0.84, 0.91)	< 0.0001
Age	–	–	1.01 (1.00, 1.01)	1.01 (1.01, 1.01)	1.00 (1.00, 1.01)	< 0.0001
Marital status	–	–				
Married	–	–	ref	ref	ref	
Single	–	–	1.19 (1.15, 1.24)	1.20 (1.15, 1.25)	1.21 (1.16, 1.26)	< 0.0001
Widowed	–	–	1.18 (1.12, 1.23)	1.18 (1.13, 1.24)	1.24 (1.16, 1.32)	< 0.0001
Divorced/separated	–	–	1.15 (1.10, 1.21)	1.15 (1.10, 1.21)	1.20 (1.13, 1.26)	< 0.0001
Not known	–	–	0.93 (0.81, 1.07)	0.94 (0.82, 1.07)	0.97 (0.83, 1.13)	0.714
IRSD						
1st quintile - most disadvantaged	–	–	ref	ref	ref	
2nd quintile	–	–	0.95 (0.91, 0.99)	0.95 (0.91, 0.99)	0.95 (0.90, 1.00)	0.061
3rd quintile	–	–	0.94 (0.90, 0.99)	0.94 (0.90, 0.99)	0.94 (0.90, 0.99)	< 0.05
4th quintile	–	–	0.92 (0.87, 0.97)	0.93 (0.87, 0.98)	0.94 (0.87, 0.98)	0.051
5th quintile – least disadvantaged	–	–	0.88 (0.82, 0.94)	0.88 (0.82, 0.94)	0.80 (0.74, 0.87)	< 0.0001
ARIA						
Highly Accessible	–	–	ref	ref	ref	
Accessible	–	–	0.93 (0.89, 0.97)	0.93 (0.89, 0.97)	0.94 (0.90, 0.98)	> 0.01
Moderately Accessible	–	–	0.88 (0.83, 0.93)	0.88 (0.83, 0.93)	0.86 (0.81, 0.92)	< 0.0001
Remote / Very Remote	–	–	0.67 (0.61, 0.73)	0.67 (0.61, 0.73)	0.65 (0.59, 0.72)	< 0.0001
Charlson Index score						
0	–	–	ref	ref	ref	
1	–	–	1.43 (1.38, 1.49)	1.43 (1.38, 1.49)	1.46 (1.40, 1.52)	< 0.0001
2+	–	–	1.63 (1.57, 1.70)	1.63 (1.57, 1.69)	1.69 (1.62, 1.77)	< 0.0001

^a^RRs are adjusted for all variables given in the table

## Discussion

This paper provides unique data on unplanned hospital readmission or death over a nine year period amongst a large cohort of Aboriginal and non-Aboriginal patients with chronic disease. To our knowledge, such an overview of unplanned readmission by Aboriginal people with chronic disease has not been undertaken before in Australia.

Aboriginal people with chronic disease have a significantly higher risk of an unplanned readmission or death within 28 days of discharge compared with non-Aboriginal people. This higher rate of unplanned readmission or death has remained unchanged over the nine year period examined. Direct comparisons of our estimates with other studies are challenging because of a paucity of comparable data analyses for unplanned readmissions in Aboriginal Australians with chronic disease. However when considering readmissions for any-cause, our findings are consistent with NSW government data which reports significantly higher rates of all-cause (medical and surgical) unplanned readmissions rates between Aboriginal and non-Aboriginal people, and that this rate has not significantly changed from 2005 to 2011 [9]. However the chronic disease readmission rates reported in our analysis are higher compared to readmissions for any cause. Our findings are consistent with other broader analyses of hospitalisation patterns among Aboriginal people with chronic disease which also report significantly higher rates of unavoidable or potentially preventable hospitalisations in Aboriginal with chronic disease compared with non-Aboriginal people [23, 24]. Yet these studies do not consider unplanned readmissions which measure a distinctly different indicator compared to unavoidable hospitalisations which generally reflects sub-optimal community health care, compared to unplanned readmissions which reflect a combination of poor hospital care as well as poor community follow up.

Our findings showed that unplanned readmission or death in Aboriginal people remained significantly higher than the non-Aboriginal rates, even once adjusted for sociodemographic, disease and admission variables, and for potential selection bias. The fact that Aboriginal status remains a significant risk factor, even after accounting for other variables, is consistent with chronic disease preventable hospitalisation studies in Aboriginal people, [23, 25] and the international literature which shows significant associations with ethnicity and readmission even after adjusting for sociodemographic or disease factors [26–28]. However considering the socio-demographic profile of Aboriginal patients with chronic disease is informative for program planning. Our study found a higher proportion of Aboriginal patients were female, younger, more likely to be single, live in the most disadvantaged and remote areas of NSW. This difference in socio-demographic profile should be considered in strategies aimed at reducing unplanned readmissions in Aboriginal people with chronic disease.

The fact that the significant difference in readmission or death rates has consistently remained over the nine years highlights the ongoing disparity between Aboriginal and non-Aboriginal health outcomes. Therefore, further targeted programs need to address the gap in effective care for high risk Aboriginal patients with chronic disease. The high prevalence of chronic diseases among patients, particularly the presence of multiple comorbidities in adults, requires intensive case management in both hospital and community settings, to ensure follow up post discharge is adequately conducted [29]. Qualitative work on the effectiveness of discharge planning and post-acute care for Aboriginal patients in improving health outcomes such as readmission, suggests good outcomes are dependent on the availability, knowledge and use of post-acute services and better access to primary health care [30]. One current NSW Health program targets Aboriginal patients recently discharged from hospital with a chronic disease and provides telephone follow-up within 48 h. It demonstrated a significant decrease in emergency department presentations, but not in unplanned readmissions, in Aboriginal people who received the follow up compared with eligible Aboriginal people who did not [31]. Further research is needed to determine the types of interventions that are effective in reducing unplanned readmissions in Aboriginal people with chronic disease.

Our finding that length of stay was shorter for Aboriginal people compared to non-Aboriginal differs to other studies. Banham and colleagues in their study of potentially preventable hospitalisations in Aboriginal people with chronic disease report higher length of stay compared to non-Aboriginal people [23]. Although shorter length of stay is often considered more efficient, it may indicate either a higher risk of discharge against medical advice in this group of patients, or that they are not receiving the sufficient care resulting in poorer health outcomes and increased risk of readmission [32, 33]. Further research should investigate length of stay in Aboriginal people with chronic disease.

### Limitations

Study findings should be considered in light of several limitations. There may be an underrepresentation in unplanned readmission rates due to underreporting of Aboriginality in hospital data, and therefore caution is needed in interpreting all hospital-level data for Aboriginal people. Further, not all data on non-Aboriginal separations is included in this analysis, only a sample of non-Aboriginal separations were included who met the eligibility criteria (compared to all Aboriginal cases where all cases meeting the criteria were selected). Therefore it is possible that the non-Aboriginal sample is not representative of all non-Aboriginal people meeting the study eligibility criteria.

We could not exclude the possibility that a selection bias was induced through selecting only those that survived the admission. Our propensity score analysis attempted to resolve this by weighting the analysis sample such that the distribution of selection confounders was similar to those that died during admission, however there may have been unmeasured confounders which biased the results. Caution should also be used in interpreting data on unplanned readmission as these data do not differentiate between avoidable and unavoidable unplanned readmissions, and therefore inevitably includes some readmissions which are appropriate and unavoidable. Finally, while it is likely that some patients had readmissions prior to 2005, our retrospective cohort study design allows us to only examine admissions within a defined time period only.

## Conclusion

Aboriginal people with chronic disease had a significantly higher risk of unplanned readmission or death compared with non-Aboriginal people, and there has been no significant change over the nine year period. It is critical that effective interventions to reduce unplanned readmissions for Aboriginal people are identified.

## Additional file

Additional file 1: ICD-10 codes. A list of ICD-10 codes for eligible chronic diseases for this retrospective cohort study. (DOCX 18 kb)

## Abbreviations

ABS: Australian Bureau of Statistics
APDC: NSW Admitted Patient Data Collection
ARIA: Accessibility/Remoteness Index of Australia
CCI: Charlson Co-morbidity Index
CI: confidence interval; RR: relative risk
EDDC: Emergency Department Data Collection
GLM: generalised linear model
IRSD: Index of Relative Socio-economic Disadvantage
NSW: New South Wales
RBDM: NSW Registrar of Births, Deaths and Marriages

## Footnotes

1 The importance of using correct, respectful and appropriate terminology for Aboriginal and Torres Strait Islander people is acknowledged. In keeping with NSW Health recommendations and acknowledging that Aboriginal people are the original habitants of NSW, the term Aboriginal people will be used (NSW Health “Communicating positively: A guide to appropriate Aboriginal terminology”, NSW Department of Health 2004).
